# Classification of Decayed Cavities in the Teeth

**Published:** 1856-12

**Authors:** J. Taft


### CLASSIFICATION OF DECAYED CAVITIES IN THE TEETH.

BY J. TAFT.

The following classification of decayed cavities of the teeth is presented, not because it is perfect; but we have found it valuable in many respects, and especially so to the student.

It is desirable to have the most systematic arrangement of all subjects pertaining to dental practice.

This classification is based on the position of the cavities, and the extent of the decay, but principally on the former.

The *classes* arise from the position, and the *modifications* from the extent of the decay.

The classification begins with those cavities most easily approached and operated upon in filling; and as by position the cavities are more difficult to operate upon, they are ranked into the successive classes.

The most simple of these decays, respectively, constitutes the first form, in each class. And then, as the decay becomes more extended, and renders the difficulty of operating on a cavity of any given class quoted, the various modifications arise.

The classification exhibits the *position* of the decay. The modifications the *extent* of the decay.

FIRST CLASS.—Central crown cavities in the molars and bicuspids.

1st Mod.—Extension along one fissure.

2d Mod.—Extension along two or more fissures.

3d Mod.—Two cavities upon the same crown, in close proximity, that may be formed into one cavity for filling.

SECOND CLASS.—Cavities upon the buccal and palatal surfaces of the molars and bicuspids; and labial and palatal surfaces of the canines and incisors.

1st Mod.—The cavity extending below the margin of the gums.

2d Mod.—Extension of the decay till a portion of the crown surface is involved.

THIRD CLASS.—Anterior approximal cavities of the bicuspids and molars.

1st Mod.—Extension of the decay toward the neck of the tooth, extending beyond the termination of the enamel.

2d Mod.—Extension of decay till the loss of a portion of the grinding or crown surface is effected.

FOURTH CLASS.—Approximal cavities of the incisors and canines.

1st Mod.—Palatal wall of the cavity broken away.

2d Mod.—Labial wall broken away.

3d Mod.—The cavity at the point of the tooth terminating at the surface.

4th Mod.—The borders of the cavity very thin, and the cavity of a conical form.

FIFTH CLASS.—Posterior approximal cavities of the molars and bicuspids.

The modifications of this class are the same as those of class third.

Modifications common to all the classes:—1st. Superficial cavity, and a large orifice.

2d. Deep cavity, and small orifice.

Modification common to classes three, four, and five:—A transverse extension of the decay, round one or more angles of the tooth, under the termination of the enamel.

This classification is altogether irrespective of pathological conditions.

By studying carefully these classes and their modifications, or some such systematic arrangement, and the best mode of operating upon each individual cavity, a more perfect comprehension of the subject will be attained, than by any examination of the subject without such a division.

